# Changes in subdomains of non-organized physical activity between childhood and adolescence in Australia: a longitudinal study

**DOI:** 10.1186/s12966-022-01311-2

**Published:** 2022-06-25

**Authors:** Byron J. Kemp, Anne-Maree Parrish, Marijka Batterham, Dylan P. Cliff

**Affiliations:** 1 Early Start, University of Wollongong, Wollongong, NSW Australia; 2 School of Education, Faculty of the Arts, Social Sciences and Humanities, University of Wollongong, Wollongong, NSW Australia; 3 School of Health and Society, Faculty of the Arts, Social Sciences and Humanities, University of Wollongong, Wollongong, NSW Australia; 4 Illawarra Health and Medical Research Institute, University of Wollongong, Wollongong, NSW Australia; 5 Statistical Consulting Centre, National Institute for Applied Statistical Research Australia, University of Wollongong, Wollongong, NSW Australia

**Keywords:** Non-organized physical activity, Active play, Exercise, Sport, Child, Adolescent, Health promotion, Social marketing, Longitudinal

## Abstract

**Background:**

Physical activity (PA) participation among youth tends to be insufficient and is prone to decline with age. In Australia, this decline has been shown to particularly occur in the domain of non-organized PA (e.g. active play and informal sport) between childhood and adolescence. However, information about changes in more specific groupings of activities within non-organized PA (i.e. subdomains) is needed, as this could support more targeted intervention strategies. This study aimed to investigate changes in the duration of specific subdomains of non-organized PA between late childhood (10–11 years) and early adolescence (12–13 years) in Australia, as well as whether these changes are moderated by sex.

**Methods:**

Data were sourced from Waves 6 and 7 of the Longitudinal Study of Australian Children (*n* = 3614). Youth time-use diaries (24-h) were used to measure the duration of eight subdomains of non-organized PA at both waves (athletics/gymnastics, ball sports, cycling/motor/roller sports, fitness/gym/exercise, martial arts/dancing, water/ice/snow sports, active play and other outdoor/nature PA). Multilevel mixed modelling was used to explore longitudinal changes between waves and the potential moderation effect of sex.

**Results:**

Active play declined the most of all subdomains (β = –20.5 min/day; 95% CI = –23.4, –17.6, *p* < 0.001). A smaller decline was observed in the subdomain of non-organized ball sports (β = –4.1 min/day; 95% CI = –5.9, –2.3, *p* < 0.001). Other subdomains remained stable or had only very small changes in participation. The decline in active play was moderated by sex, with a steeper decline among girls. No other notable moderation effects were observed.

**Conclusions:**

Future studies may seek to explore and test the acceptability of PA promotion strategies to encourage active play participation, such as ‘reframing’ childhood play activities to be appropriate for adolescents. Such studies might particularly seek the perspectives of girls in the transition to adolescence.

**Supplementary Information:**

The online version contains supplementary material available at 10.1186/s12966-022-01311-2.

## Background

Regular participation in physical activity (PA) has been linked with many health benefits for children and adolescents [1]. However, PA participation among youth has been frequently reported to be insufficient and prone to decline with age [2]. Recent studies have investigated whether this decline occurs in particular domains of PA [3], and a national Australian longitudinal study has reported that the domain of non-organized PA was particularly prone to decline between 10-11y and 12-13y [4]. Non-organized PA includes a range of activities that are relatively unstructured, freely-chosen and occurring for their own sake (e.g. playground games, informal ball games, dancing for fun, playing in a pool) [5]. Yet despite the commonalities between activities in this domain of PA, there may also be *intra-domain* differences in participation. For example, informal ball games may involve a greater degree of skill compared with playground games [6]. Additionally, post-pubescent girls might be more self-conscious about swimming compared with less physically-revealing activities [7]. Therefore, specific non-organized activities may be prone to change at different ages and these changes may vary by sex. An exploration of changes in more specific groupings of activities within non-organized PA (i.e. subdomains) could yield useful information to support intervention strategies during the transition from childhood to adolescence in the Australian context.

There is limited longitudinal evidence about subdomain-level changes in non-organized PA between childhood and adolescence. In particular, no known studies have explored these changes in the Australian context. Longitudinal studies conducted elsewhere have reported declines in types of non-organized PA during this stage of life, such as in active play (e.g. jump rope, playground games) [8–10] and walking for exercise/leisure [10, 11]. Longitudinal declines in specific sports have also been reported between childhood and adolescence, such as basketball [8, 10, 12], hockey [8, 9], football [8, 10] and swimming [8, 12]. However since these studies did not differentiate between domains of PA, it is unknown if these changes occurred as part of organized club sports (organized PA) or in the context of informal, self-organised activities (non-organized PA). Similarly, some studies have reported declines in general exercise/conditioning [8, 10] and running/jogging [8, 10, 12] but they did not differentiate between exercise classes/clubs (organized PA) and self-directed exercise (non-organized PA). Finally, studies have reported declines in cycling [8, 10, 11] and rollerblading/skating [8, 9, 12] but these also could have occurred in multiple domains, namely non-organized PA and/or active transport.

There is also limited evidence suggesting that longitudinal changes in subdomains of non-organized PA may differ by sex between childhood and adolescence. Among the studies mentioned above, only three studies reported results separately for boys and girls [8, 10, 12]. These studies reveal some similarities and differences by sex. Two studies indicated that outdoor play/playground games and rollerblading/skating were likely to decline among both boys and girls [8, 10]. Cycling also appeared to decline among both sexes in USA [12], although this activity was particularly likely to decline among girls in Canada [8]. In the USA, running/jogging appeared more likely to decline among boys between approximately 10-11y and 12-13y in one study [10] but were more prone to decline among girls between 11-14y and 12-15y in another [12]. In the USA, baseball was more likely to decline in prominence among boys, while swimming and dancing was more likely to decline among girls [12]. Overall, the results of these studies indicate that some types of PA are prone to decline among both sexes between childhood and adolescence, while other types of PA may particularly decline among boys or girls. However, the specific types of PA in question appear to vary depending on age, geographic location and/or study methodology. In addition, it is unclear how these findings might translate to the context of non-organized PA because none of these studies differentiated between domains of PA.

In summary, the extant literature relevant to this study appears to be limited in multiple ways. Firstly, few existing studies have differentiated between domains of PA. This means it is difficult to comprehensively and directly compare changes in subdomains of non-organised PA based on these studies. In addition, due to geographic and measurement differences between studies, it is important to examine changes in several subdomains of non-organized PA concurrently in the same sample. To the authors’ knowledge, no previous studies have explored changes in several types/subdomains specific to non-organised PA between childhood and adolescence, nor have changes by sex been investigated.

Therefore, this study aimed to investigate two research questions: (1) How does the duration of subdomains of non-organized PA change between late childhood (10-11y) and early adolescence (12-13y) in Australia; and (2) Are these changes moderated by sex?

## Materials and methods

### Setting and procedures

This study used data from the Baby (B) cohort of the Longitudinal Study of Australian Children (LSAC), a longitudinal research project managed by the Australian Department of Social Services (DSS) [13]. The overall aim of the LSAC project is to “identify policy opportunities for improving support for children and their families, and identifying the opportunities for early intervention” [13]. Data are publicly available via application [14]. LSAC data collection procedures were approved by the Australian Institute of Family Studies Ethics Committee, and participants provided informed consent [15]. The use of data in the present study has been approved by the University of Wollongong Human Research Ethics Committee (HREC 2022/046).

### Participants

LSAC includes two cohorts of participants who were recruited in 2004. The Kindergarten (K) cohort were recruited at 4-5y and the Baby (B) cohort were aged 0–12 months at baseline [13]. Participants in both cohorts were recruited from the Australian Medicare database via a two-stage clustered design, involving the random selection of postcodes then families [13]. Both cohorts were designed to be nationally-representative samples of the Australian population, with stratified selection being used to ensure proportional inclusion of members of each Australian state and territory across major city and regional/remote areas [16]. Both cohorts have been followed-up every two years to date, with a variety of follow-up methods being employed including mail, telephone and in-person visits [13]. The two cohorts also follow a sequential design, where the B cohort data collection tends to roughly resemble what was collected from K cohort participants 4 years earlier. However, data collection improvements sometimes result in variation in the measures collected from each cohort at the same age.

Although previous analyses of non-organized PA have been conducted with K cohort data [4], we opted to use B cohort data in the present study due to two main advantages of this cohort. Firstly, the B cohort datasets provided more granular time-use data which supported the analysis of changes at the subdomain level (previous analyses of K cohort data were restricted to the entire domain of non-organized PA). Secondly, B cohort time-use data have been collected more recently. B cohort participants were 10-11y in 2014 (Wave 6) and 12-13y in 2016 (Wave 7), whereas the equivalent K cohort data were collected in 2010 (Wave 4) and 2012 (Wave 5). A disadvantage of using B cohort data was a higher rate of attrition, as data had been collected two waves later in the study. Compared with K cohort data used previously [4], there were 539 fewer participants in the B cohort at 10-11y and 602 fewer participants at 12-13y. However, despite the higher rate of dropout in the B cohort, the demographics of both cohorts at 10-11y and 12-13y appeared similar (see Additional file 1). Importantly, the B cohort did not have a higher rate of dropout among key population groups that are prone to attrition, such as participants from lower socioeconomic backgrounds, participants from Aboriginal and Torres Strait Islander backgrounds or those who spoke languages other than English at home [17]. Therefore, using B cohort data in the present study was considered a net benefit overall.

As mentioned above, the present use of LSAC data follows on from a previous study which used data from the Kindergarten (K) cohort of LSAC. This previous study explored changes in non-organized PA, organized PA, active transport and active chores/work between childhood and adolescence [4]. Given the change to the B cohort in the present study, key aspects of this study have been replicated as detailed in Additional file 1. This revealed mostly the same findings as the previous study, namely a large decline in non-organized PA, a stable trend in organized PA and a slight increase in active transport between 10-11y and 12-13y. The main difference from the previously reported findings was a stable trend in active chores/work between waves in the B cohort, compared with the slight increase observed in K cohort data [4].

### Physical activity measures

Participation in subdomains of non-organized PA was measured using time-use diaries (TUDs) completed at 10-11y and 12-13y. This is the longest time-series that could be used for this study because reduced ‘light diaries’ were used prior to Wave 6 and free-text descriptions of activities were not included after Wave 7. Participants recorded activities in their own words using paper diaries over the 24-h period on the day before their interview [18]. Participants who attended school on this day were instructed to record activities conducted during school break-times but not during lessons (such as physical education (PE) lessons). Diary entries were then coded by interviewers during the home visit using a predetermined coding framework [18]. Interviewers were trained to prompt the child for additional information during this process (including filling gaps in the diary) [18].

**Fig. 1:**
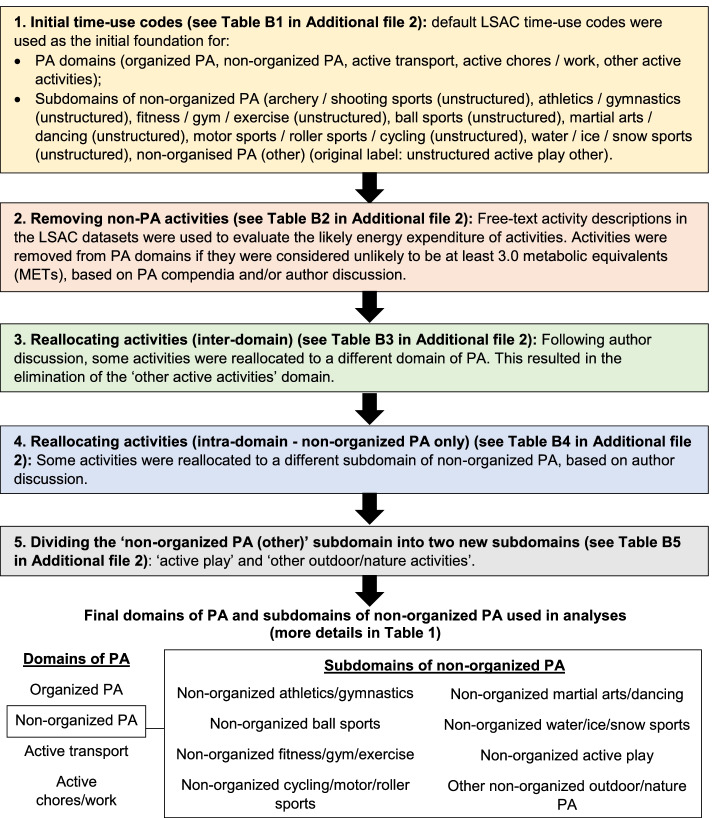
Overview of the process used to clean TUD data based on LSAC activity descriptions

TUD data were processed in a similar manner as in previous research [4]. However, an additional quality improvement process was also conducted using free-text descriptions of TUD activities (see Fig. 1). This process was not undertaken previously because free-text descriptions were only available in B cohort data. At the beginning of the process, default LSAC TUD codes were used as the foundation for domains of PA and subdomains of non-organized PA (see Fig. 1). Free-text descriptions were then evaluated to determine if activities should be considered moderate-to-vigorous physical activity (MVPA). The threshold of 3.0 metabolic equivalents (METs) was used as the minimum cut-off for MVPA as defined by the World Health Organization [19]. Activities were evaluated based on (in order): the current or previous youth energy expenditure compendia [20, 21]; the current adult compendium [22]; or author consensus. Where compendia provided MET scores for different intensities of the same activity, the score for the ‘medium’ or ‘moderate’ intensity was taken. Some activities were removed from domains of PA via this process, as listed in Additional file 2 (e.g. removing ‘billiards’ from non-organized PA). The authors also determined that some activities should be recoded to a different domain of PA (e.g. unstructured wood chopping was moved from non-organized PA to active chores/work). Some activities were also reallocated to a different subdomain of non-organized PA (e.g. walking for exercise was reallocated from non-organized PA (other) to non-organized fitness/gym/exercise). A full list of reallocations is provided in Additional file 2.

The final stage of processing TUD data involved dividing the relatively heterogeneous subdomain of ‘non-organized PA (other)’ into two smaller categories to support more meaningful analysis. In particular, active play activities were identified and separated from other types of PA, given that a declining pattern in active play was one of the more consistent findings in the limited amount of background literature on this topic [8–10]. Activities in this subdomain were coded as active play if: (i) they were likely to satisfy Pellegrini and Smith’s criteria for physically active play (occurring for its own sake and occurring in a ‘playful context’ [23]); and (ii) if they were consistent with at least one example of physical play included in the Tool for Observing Play Outdoors, such as gross motor play (e.g. running, jumping, throwing, kicking) or vestibular play (e.g. balancing, skipping, sliding, using monkey bars) [24]. The activity ‘unstructured active play (not further defined)’ was also allocated to active play [25]. The remaining activities that were not included in active play were grouped together into an eighth subdomain labelled ‘other outdoor/nature activities’. The final subdomains of non-organized PA are described in Table 1 and more details are available in Additional file 2.

**Table 1:** Activities included in the final subdomains of non-organized PA used in the present study^a^

Subdomain of non-organized PA	Activities included in the subdomain
Non-organized athletics/gymnastics	Acrobatics, Cheerleading, Gymnastics
Non-organized ball sports	American football, Australian Rules football/AFL, Badminton, Baseball, Basketball, Carpet bowls/lawn bowls, Football (other), Golf, Handball, Netball, Outdoor cricket, Outdoor hockey, Outdoor soccer, Oztag, Putt-putt golf/minigolf, Rugby league, Rugby union, Softball, T ball, Table tennis/ping pong, Tennis, Tenpin bowling, Touch football, Ultimate Frisbee, Volleyball/beach volleyball, Non-organised ball sports (other)
Non-organized cycling/motor/roller sports	Bike riding/cycling, BMXing/mountain biking, Dirt/trail biking motorised, Roller blading/skating, Scooter (ride unstructured), Skateboarding/Skate park, Non-organised cycling/roller sports (other)
Non-organized fitness/gym/exercise	Calisthenics, Circuits, Exercise biking, Exercising, Gym workouts, Jogging, Running for exercise, Treadmill activities, Walking (unstructured exercise), Weightlifting
Non-organized martial arts/dancing	Boxing, Dancing (other), Martial arts (other), Wrestling, Non-organised martial arts/dancing (other)
Non-organized water/ice/snow sports	Bodyboarding, Canoeing, Ice skating, Sailing, Snowboarding, Snow skiing, Surf sport/Surfing, Swimming, Water-skiing, Non-organised water/ice/snow sports (other)
Non-organized active play	Flying disc games/boomerang throwing, Flying kites, Hide and seek, Hula hoop, Kicking ball/ball games, Play on play equipment (playground/play on play equipment, going to the park), Rope skipping/skipping, Slip n slide, Throwing ball against wall, Tips/chasing/running around, Trampolining, Water fight, Unstructured active play (not further defined)
Non-organized other outdoor/nature PA	Archery (unstructured), Bush walking, Exploring/Going sightseeing, Fishing, Horse riding, Yabbying

^a^More details are included in Additional file 2, including rationales and MET scores

### Other measures

Wave of measurement (W6/W7) was the main explanatory variable in this study. Sex was also included in models as a potential moderator (male/female). Models were also controlled for two potential confounding variables: season of measurement and whether the child attended school on the day of TUD completion (yes/no). Season was derived from the interview month and school attendance was included as a variable in LSAC datasets. Missing data for school attendance was imputed using the ‘school lessons’ code in the TUD (it was assumed that children attended school if this code was used).

### Analysis

Data were processed using SPSS version 25 (IBM Corporation, Armonk, NY, USA) and statistical analyses were performed using Stata 15 (StataCorp, College Station, TX, USA).

As with K cohort data [4], frequency histograms revealed that respondents tended to round their TUD entries to the nearest 5 min. Thus for consistency, domains of PA and subdomains of non-organized PA were rounded to the nearest 5 min for all cases. In addition, robust standard errors were used in models because some heteroscedasticity was observed in residual versus fitted plots. The LSAC Wave 1 and 6 population data weights were applied to reduce bias associated with attrition and improve the representativeness of the data.

Longitudinal changes in each subdomain of non-organized PA were tested using separate multilevel mixed-effects models. All available data were included in models, as multilevel modelling does not require complete cases for every time-point [26]. Preliminary models tested the effect of wave on each subdomain of non-organized PA (level 1), nested within individuals (level 2). Models were then adjusted for season of measurement and whether the child attended school on the day of TUD completion. Finally, post-hoc models tested interactions between wave and sex.

## Results

A total of 3764 participants took part in the W6 face-to-face interview and 3614 of these participants provided complete TUD data for at least one wave and were included in the models (96% of the total W6 sample). Among these, 3455 participants provided complete TUD data for W6 and 2971 participants provided complete TUD data for W7. Participant characteristics for those with valid TUD data at each wave are shown in Table 2. A similar proportion of those with valid TUD data at W7 were male compared with 12–13 year-olds in the 2016 Australian Census (51.0% at W7 versus 51.4% nationally) [27]. The analytic sample somewhat overrepresented participants who lived in regional or remote areas (36.3% at W7 versus 30.4% nationally) and underestimated Aboriginal and Torres Strait Islander participants (2.3% at W7 versus 5.1% nationally) and those who spoke languages other than English at home (8.8% at W7 versus 16.9% nationally) [27]. These discrepancies were all somewhat attenuated via data weighting (see Table 2, footnote c).

**Table 2:** Characteristics of the sample with valid TUD data in each wave, unweighted LSAC data

	**Wave 6** **10-11y, 2014** **(*n* = 3455) **^**a**^	**Wave 7** **12-13y, 2016** **(*n* = 2971) **^**b**^
Males, n (%)	1756 (50.8%)	1514 (51.0%)^c^
Age, mean (SD)	10.4 (0.5)^d^	12.4 (0.5)^d^
Family socioeconomic position index, mean (SD) ^e^	0.0 (1.0)	0.0 (1.0)
Speaks language other than English at home, n (%)	291 (8.4%)	262 (8.8%)^c^
Aboriginal or Torres Strait Islander, n (%)	92 (2.7%)	67 (2.3%)^c^
Lives in regional or remote area, n (%)	1251 (36.2%)	1077 (36.3%)^c^
Attended school on day of TUD, n (%)	1683 (48.7%)	1548 (52.1%)
Season of measurement, n (%)		
Summer	69 (2.0%)	256 (8.6%)
Autumn	846 (24.5%)	439 (14.8%)
Winter	1373 (39.8%)	1626 (54.8%)
Spring	1162 (33.7%)	649 (21.9%)

*LSAC* Longitudinal Study of Australian Children, *n* number of participants, % proportion of sample, *SD* standard deviation, *TUD* time use diary. Percentages may add to more than 100% due to rounding

^a^Variable-specific missing data for Wave 6: socioeconomic position index (*n* = 29), season of measurement (*n* = 5), main language spoken at home (*n* = 1)

^b^Variable-specific missing data for Wave 7: socioeconomic position index (*n* = 14), season of measurement (*n* = 1)

^c^Weighted proportions for Wave 7 (comparison with national Census data [27]): 51.1% were male (Census: 51.4%), 12.5% spoke languages other than English at home (Census: 16.9%), 2.8% were of Aboriginal or Torres Strait Islander origin (Census: 5.1%) and 33.2% lived in regional or remote areas (Census: 30.4%)

^d^Of the participants with valid data in both waves, the average time between waves was 24.5 months (SD=3.0, *n* = 2812)

^e^The socioeconomic position index was z-scored and ranged from -5.5 to 2.7 in Wave 6 and from -7.4 to 2.7 in Wave 7

**Fig. 2:**
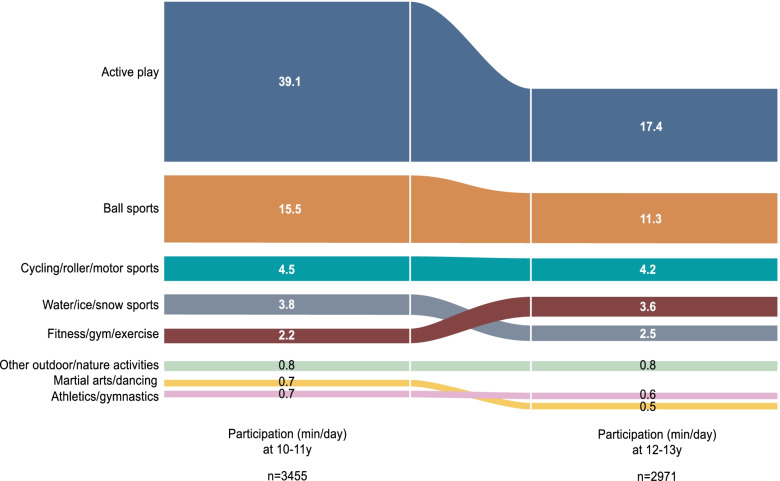
Participation in subdomains of non-organised PA at 10-11y and 12-13y, weighted LSAC data, B cohort

Table 3 and Fig. 2 outline the longitudinal trends in subdomains of non-organized PA between 10-11y and 12-13y (see also Additional file 3). The vast majority of the decline between 10-11y and 12-13y occurred in the subdomain of active play, which declined by an average of 21 min/day between waves (β = –20.5; 95% CI = –23.4, –17.6; *p* < 0.001). Relatively small declines were also observed in the subdomains of non-organized ball sports (β = –4.1; 95% CI = –5.9, –2.3; *p* < 0.001) and water/ice/snow sports (β = –1.3; 95% CI = –2.3, –0.3; *p* = 0.01). The only subdomain that increased in participation between 10-11y and 12-13y was fitness/gym/exercise, which increased by an average of one minute per day (β = 1.3; 95% CI = 0.4, 2.2; *p* = 0.003). The subdomains of non-organized cycling/roller/motor sports, athletics/gymnastics, martial arts/dancing and other outdoor/nature activities remained stable between 10-11y and 12-13y.

**Table 3:** Participation in subdomains of non-organized PA (minutes/day) by wave, weighted LSAC data, B Cohort

	**Participation, min/day** **Mean (SD)**		**Unadjusted models – fixed effect of wave **^**a**^		**Adjusted models – fixed effect of wave **^**a**^^**b**^	
	**10-11y (w6) ** ***n* = 3455**	**12-13y (w7)** ***n* = 2971**	**β (95% CI)**	***p* value**	**β (95% CI)**	***p* value**
**Subdomains of non-organized PA**						
Active play	39.1 (78.6)	17.4 (51.6)	-21.7 (-24.6, -18.7)	<0.001	-20.5 (-23.4, -17.6)	<0.001
Ball sports	15.5 (41.6)	11.3 (40.4)	-4.2 (-6.0, -2.3)	<0.001	-4.1 (-5.9, -2.3)	<0.001
Water/ice/snow sports	3.8 (25.4)	2.5 (23.0)	-1.3 (-2.4, -0.1)	0.03	-1.3 (-2.3, -0.3)	0.01
Cycling/roller/motor sports	4.5 (28.7)	4.2 (28.3)	-0.4 (-1.7, 1.0)	0.62	-0.2 (-1.6, 1.2)	0.77
Fitness/gym/exercise	2.2 (14.7)	3.6 (18.7)	1.3 (0.5, 2.2)	0.002	1.3 (0.4, 2.2)	0.003
Athletics/gymnastics	0.7 (9.3)	0.6 (18.0)	-0.1 (-0.8, 0.6)	0.82	0.0 (-0.9, 0.9)	0.98
Martial arts/dancing	0.7 (8.2)	0.5 (7.2)	-0.2 (-0.6, 0.1)	0.22	-0.2 (-0.6, 0.1)	0.18
Other outdoor/nature activities	0.8 (12.1)	0.8 (13.9)	0.1 (-0.6, 0.7)	0.79	0.2 (-0.4, 0.8)	0.52

*PA* physical activity, *LSAC* Longitudinal Study of Australian Children, *SD* standard deviation, *β* model coefficient, *CI* confidence interval, *W* wave

^a^Multilevel mixed models (*n *= 3614)

^b^Adjusted for season and school attendance on the day of TUD completion

**Fig. 3:**
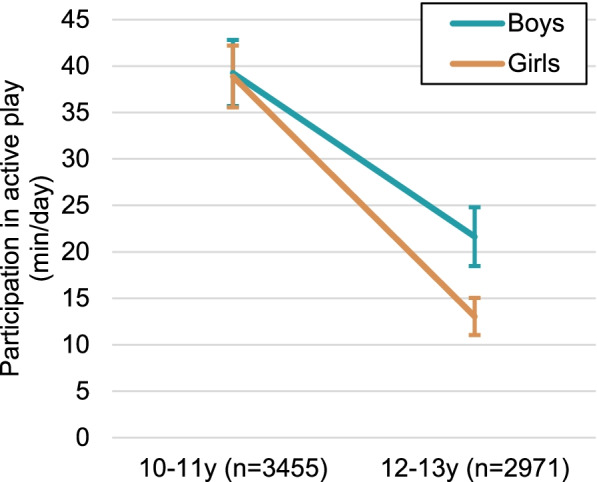
Participation in active play at 10-11y and 12-13y by sex, weighted LSAC data, B Cohort

Active play was the only subdomain of non-organized PA that exhibited a notable moderation effect by sex. As shown in Fig. 3, a steeper decline between 10-11y and 12-13y occurred among girls compared with boys (β = –8.2, 95% CI = –13.9, –2.4; *p* = 0.006). Additional file 4 provides the results of all moderation tests.

## Discussion

The present study aimed to investigate two research questions: (1) How does the duration of specific subdomains of non-organized PA change between 10-11y and 12-13y in Australia; and (2) Are these changes moderated by sex? Active play declined the most of all subdomains (–21 min/day) and this was moderated by sex, with a steeper decline evident among girls. Smaller declines in participation were observed in the subdomains of non-organized ball sports (–4 min/day) and water/ice/snow sports (–1 min/day), and a slight increase was observed in non-organized fitness/gym/exercise (+ 1 min/day). These changes were not notably moderated by sex. The other subdomains of non-organized PA remained stable and did not exhibit notable moderation effects between 10-11y and 12-13y.

The substantial decline in active play observed in the present study was consistent with previous longitudinal studies that reported declines in outdoor play [8], playground games [10], skipping/jump rope [8, 9] and playing with younger children during late childhood and adolescence [10]. These activities are all somewhat ‘childish’ in nature, which is consistent with a theme in a recent qualitative study describing dropout from non-organized PA during the transition from childhood to adolescence [28]. In a sense this may be seen as a somewhat axiomatic finding – merely reflecting natural developmental changes in children’s leisure-time preferences as they age [6]. However, it also reflects the evolving landscape of social norms and expectations during the transition to adolescence [29], which may influence adolescents’ future PA participation. Adolescents often withdraw from active play due to fears of peer judgement [28, 30] and instead begin engaging in PA for more utilitarian reasons, such as physical appearance or health benefits [28]. This may explain the slight increase in the ‘fitness/gym/exercise’ subdomain between 10-11y and 12-13y in the present study. Additionally, some adolescents begin to view PA participation as being almost exclusively associated with sport during this stage of life [31]. This can pose a problem for youth who view themselves as ‘non-sporty’, such as those who are less confident in their physical skills [32]. ‘Non-sporty’ youth may even conclude that PA is not compatible with their identity and this assumption may persist into later life [28]. As a result, ‘non-sporty’ youth may be left with few socially-sanctioned forms of PA that are compatible with their interests and identities.

The decline in active play between 10-11y and 12-13y in the present study was steeper among girls compared with boys. This finding differed from the results of previous studies, where declines in playground games/outdoor play were reported among both boys and girls [8, 10]. However, it is difficult to directly compare these findings because previous studies operationalised participation in active play as a categorical outcome (yes/no) [8, 10], whereas the present study analysed changes in the duration of participation (min/day). This being said, a steeper decline in active play participation among girls between childhood and adolescence is reasonable to expect, as the onset of puberty tends to occur earlier among girls compared with boys [33]. Girls often become more self-consciousness during puberty which may result in greater reluctance to engage in PA [32]. The earlier onset of puberty among girls may also lead to a greater desire to avoid ‘childish’ types of PA, as pubertal onset has been shown to promote more adult-typical behaviors [34].

This study also reported slight declines in the subdomains of non-organized ball sports and water/ice/snow sports between 10-11y and 12-13y. However, the decline in water/ice/snow sports was particularly small and may not be practically meaningful (–1 min/day). Previous studies also reported declines in ball sports such as basketball [8, 10, 12], hockey [8, 9] and football [8, 10], as well as swimming [8, 12], although these studies did not differentiate between domains of PA. The slight decline in non-organized ball sports in the present study is interesting given the previous discourse about youth increasingly associating PA with sport during adolescence [31]. One explanation may be that youth often perceive sport as becoming more serious and performance-oriented during adolescence, which may lead them to withdraw from non-organized sports if they are not confident in their physical skills [28, 32]. Another possible explanation is that the slight declines in non-organized ball sports and water/ice/snow sports may also reflect broader shifts in time-use patterns during the transition to adolescence. For example, additional barriers to PA participation arise during adolescence, such as changing leisure-time and social interests [30, 35, 36] and new responsibilities associated with school [30, 35]. Given that time is a limited resource, any increases in these alternative activities would result in less time being available for other activities, including non-organized PA participation [37].

This leads to some potential implications and directions for future research arising from this study. As time is a limited resource, future studies may draw on the social marketing concept of ‘competitor analysis’ by seeking to identify alternative activities that may take the place of subdomains of non-organized PA in the transition to adolescence [38]. For example, texting and social media use may be a particularly important alternative to certain subdomains of non-organized PA among youth [39]. The findings of such studies might eventually inform the design of interventions to appeal to the interests of certain segments of youth, such as the use of active augmented reality games (e.g. Pokémon Go) to appeal to youth who are motivated by electronic gaming [40]. Other future studies might focus on the subdomain of active play, since this subdomain was particularly prone to decline in the present study. For example, future qualitative studies might seek to involve youth in the co-design of intervention strategies to promote active play in the transition to adolescence. Such studies might focus on the perspectives of girls, since girls had a steeper decline in active play in the present study. The acceptability of certain intervention strategies may also be explored with youth, such as ‘reframing’ active play by emphasising activities that are similar to active play but with important contextual differences to reduce childish connotations (e.g. a ‘ropes course’ instead of a playground) [28, 41]. In addition, active play initiatives targeting adults have arisen organically in recent times, such as the University of Nottingham’s Society of Childhood Games, in which university students play active games such as hide and seek [42]. Future studies might test the acceptability of such initiatives among adolescent audiences.

This was the first known study to explore changes in several types/subdomains specific to non-organised PA between childhood and adolescence, and the first to explore these changes by sex. However, some limitations of this study are worth noting. Firstly, although analyses controlled for school attendance on the day of TUD completion, this may not fully account for variation in participants’ routines between days of the week. Despite this, it is noteworthy that the present study was able to broadly replicate the domain-level findings of a previous study that used 24-h TUDs in a different sample [4] (see Additional file 1), and other 24-h TUDs have demonstrated promising validity [43] and reliability [44]. Another limitation is the potential for recall bias due to the use of self-reported TUDs, as self-reported measures have been shown to overestimate PA (in potentially up to 70% of instances [45]). However, self-report methods are often needed to capture information about PA context which cannot easily be obtained via more objective methods such as accelerometry [46]. In addition, some subdomains had high counts of zero participation, due to these activities occurring infrequently in the sample (particularly non-organized athletics/gymnastics, martial arts/dancing and other outdoor/nature activities). Although the lack of participation in these subdomains is in itself relevant for health promotion, there may have been limited statistical power to detect changes in these subdomains. In addition, although the chosen population weight was considered the best available option, it did not account for non-response beyond Wave 6. This resulted in some population groups being under- or over-represented in the sample, which was only partially attenuated through the use of data weights. Participants who spoke languages other than English at home were particularly under-represented, which is likely due to LSAC being a ‘closed’ longitudinal study. No new participants have been recruited to LSAC since Wave 1, which means immigrants arriving in Australia after 2004 have not been included. These factors should be considered when applying the results of this study in the Australian context or elsewhere.

## Conclusions

This study sought to explore changes in the duration of subdomains of non-organized PA between 10-11y and 12-13y in Australia and to explore whether these changes were moderated by sex. Active play declined the most of all subdomains and this was moderated by sex, with a steeper decline evident among girls. A smaller decline in participation was observed in the subdomain of non-organized ball sports. Future studies might seek to explore and test the acceptability of PA promotion strategies, such as ‘reframing’ childhood active play to be appropriate for adolescents and seeking to tailor initiatives to appeal to the alternative interests of youth. Such studies might particularly seek the perspectives of girls in the transition to adolescence.

## Supplementary Information

**Additional file 1.** Analysis of changes in overall domains of PA between 10-11y and 12-13y. This file describes the changes in overall domains of PA between 10-11y and 12-13y using time-use data from the Baby (B) cohort of the Longitudinal Study of Australian Children (LSAC).

**Additional file 2.** Documentation of the process used to clean time-use data. This file provides further details about the process used to clean time-use data, resulting in the final subdomains of non-organized PA used in analysis.

**Additional file 3.** Participation in subdomains of non-organized PA at 10-11y and 12-13y, showing mean participation (min/day) and 95% confidence intervals (weighted LSAC data, B cohort). This file provides an additional data visualisation of the changes in mean participation in subdomains of non-organized PA at both waves, including 95% confidence intervals.

**Additional file 4.** All potential moderation effects between wave and sex on subdomains of non-organized PA (minutes/day), weighted LSAC data, B Cohort. This file provides the results from all post-hoc models used to test interactions between wave and sex for each subdomain of non-organized PA.

**Additional file 5.** STROBE Statement. Completed STROBE checklist as requested in submission guidelines.

## Data Availability

The datasets supporting the conclusions of this article are available by application via the DSS Longitudinal Studies Dataverse: http://dx.doi.org/10.26193/BAA3N6. Restrictions apply to the availability of these data, which were provided under license for the current study by the Australian Department of Social Services.

## Abbreviations

B Cohort: Baby cohort of LSAC, aged 0-1y at baseline
DSS: Australian Department of Social Services
HREC: Human Research Ethics Committee
K Cohort: Kindergarten cohort of LSAC, aged 4-5y at baseline
LSAC: Longitudinal Study of Australian Children
METs: Metabolic equivalent of task
MVPA: Moderate-to-vigorous physical activity
PA: Physical activity
PE: Physical education
TUDs: Time-use diaries
W: Wave of measurement
