# Supplementary material for: Changes in subdomains of non-organized physical activity between childhood and adolescence in Australia: a longitudinal study
Source: Int J Behav Nutr Phys Act. 2022 Jun 25;19:73. doi: 10.1186/s12966-022-01311-2 (PMC9233835; doi:10.1186/s12966-022-01311-2)
Supplement: Supplementary file 1 — Additional file 1. Analysis of changes in overall domains of PA between 10-11y and 12-13y. This file describes the changes in overall domains of PA between 10-11y and 12-13y using time-use data from the Baby (B) cohort of the Longitudinal Study of Australian Children (LSAC). [file 12966_2022_1311_MOESM1_ESM.pdf]

Changes in subdomains of non-organized physical activity between childhood and adolescence in Australia: a longitudinal study

Additional file 1: Analysis of changes in overall domains of PA between 10-11y and 12-13y

Background

This file describes the changes in overall domains of PA between 10-11y and 12-13y using time-use data from the Baby (B) cohort of the Longitudinal Study of Australian Children (LSAC). As described in the main manuscript, the purpose of this analysis was to replicate a previous study conducted using data from the Kindergarten (K) cohort of LSAC [4].

Methods

Please refer to the main manuscript for general methodological information about the LSAC study. The data described in this file were collected from B cohort participants when they were 10-11y (Wave 6, 2014) and 12-13y (Wave 7, 2016). The analysis of these data has been compared with previous findings drawn from the K cohort when they were the same ages in Wave 4 (2010) and Wave 5 (2012). As shown in Table A1, a similar number of participants were recruited to both cohorts at baseline in 2004. However, as B cohort data were collected two waves later in the study, participant attrition was higher in this cohort compared with equivalent K cohort data. Relative to the K cohort participants in the previous study [4], there were 539 fewer participants in the B cohort at 10-11y and 602 fewer participants at 12-13y [13]. Yet despite the higher rate of dropout in the B cohort, the demographics of both cohorts at 10-11y and 12-13y appeared similar. As shown in Table A2, the B cohort did not have a higher rate of dropout among key population groups that are prone to attrition, such as participants from lower socioeconomic backgrounds, participants from Aboriginal and Torres Strait Islander backgrounds or those who spoke languages other than English at home [17].

Table A1: Number of participants in the LSAC sample at baseline, 10-11y and 12-13y (B and K cohorts)

	B cohort sample n (% of Wave 1 sample)	K cohort main wave sample n (% of Wave 1 sample)	Difference (B - K)
Baseline (Wave 1)	5107 (100%)	4983 (100%)	+124
At 10-11y[a]	3455 (68%)	3994 (80%)	–539
At 12-13y[b]	2971 (58%)	3573 (72%)	–602

a. Participants aged 10-11y who were included in the present study (B cohort, Wave 6) and the previous study (K cohort, Wave 4 [4]).
b. Participants aged 12-13y who were included in the present study (B cohort, Wave 7) and the previous study (K cohort, Wave 5 [4]).

Table A2: Characteristics of participants included in the present study (B cohort) and the previous study (K cohort [4]) at 10-11y and 12-13y, unweighted LSAC data

	At 10-11y		At 12-13y	
	B cohort (n=3455)	K cohort (n=3994)	B cohort (n=2971)	K cohort (n=3573)
Sex (males), n (%)	1756 (50.8%)	2035 (51.0%)	1514 (51.0%)	1807 (50.6%)
Family socioeconomic position index, mean (SD)[a]	0.0 (1.0)	0.1 (1.0)	0.0 (1.0)	0.3 (1.0)
Speaks language other than English at home, n (%)	291 (8.4%)	313 (7.8%)	262 (8.8%)	256 (7.2%)
Aboriginal or Torres Strait Islander, n (%)	92 (2.7%)	110 (2.8%)	67 (2.3%)	78 (2.2%)
Lives in regional or remote area, n (%)	1251 (36.2%)	1406 (35.2%)	1077 (36.3%)	1289 (36.1%)

a. The socioeconomic position index is z-scored (negative values = lower socioeconomic position)

Following the data cleaning process described in the main manuscript and in Additional File 2, four domains of PA were included in the analyses (see Table A3). As part of data cleaning, one implausibly high value was removed from active transport. Analyses were conducted in a similar way as those in the main manuscript. Longitudinal changes in each domain of PA were tested in separate multilevel mixed-effects models in Stata 15 (StataCorp, College Station, TX, USA). The LSAC Wave 1 and 6 population data weights were applied to analyses. Preliminary models tested the effect of wave on each domain of PA (level 1), nested within individuals (level 2). Models were then adjusted

for season of measurement and whether the child attended school on the day of TUD completion. As consistent with the main manuscript, robust standard errors were used.

Table A3: Descriptions of overall domains of PA included in analyses

**Domain of physical activity**	**Time-use diary activities**
Organised physical activity	Athletics/gymnastics (e.g., acrobatics, athletics, cheerleading, fun runs, gymnastics, organised trampolining, sports carnivals (not further defined), track and field, triathlons)<br>Fitness / gym / exercise (aerobics, boot camp, calisthenics, exercise biking, exercising, fitness classes, gym classes/workouts, jogging/running, organised rope skipping)<br>Ball Sports (various organised ball sports including baseball, basketball, bowling, cricket, football, golf/minigolf, handball, hockey, lacrosse, netball, oztag, racquet sports, rugby, soccer, softball, sports matches, t-ball, ultimate Frisbee, volleyball),<br>Martial arts / Dancing (aikido, ballet, ballroom dancing, boxing, dancing, fencing, judo, jujitsu, karate, kickboxing, martial arts, taekwondo, wrestling)<br>Motor Sports/Roller Sports/Cycling (bike riding, BMX, mountain biking, roller sports, skateboarding)<br>Water/Ice/Snow Sports (bodyboarding, ice skating, kayaking, rowing, sailing, surf lifesaving, surfing, swimming, swimming carnival, water polo),<br>Organised team sports and training other (active club meetings e.g. scouts, air sport, bow hunting, flying disc games, horse riding, orienteering, rodeos, organised sports and training (other))
Non-organised physical activity	As described in the main manuscript
Active transport	[Travel] by bike, scooter, skateboard etc. (travel by bike/BMX, scooter, skateboard, wheelchair)<br>Travel by foot (travel by walking and running)
Active chores and work	Labourers and related workers (farm hand, labourer assistant),<br>Gardening / lawn mowing (gardening, harvesting home produce, lawn mowing, maintaining plants, outdoor /yard work, weeding, wood chopping)<br>Umpiring [paid] (umpiring)<br>Car washing [paid] (car washing),<br>Cleaning/tidying (clean bathroom/bed/bedroom/bench/floors/kitchen/laundry/ lounge room/windows, dusting, home duties/housework, mopping, move furniture, sweeping, tidying, vacuuming, washing (folding clothes))<br>Cleaning grounds/garage/shed/outside of house (clean deck/garage/outside/ shed, load/unload trailer, sweep/rake leaves)<br>Pool care (chores) (clean swimming pool/pond)<br>Design/Home Improvement (build shed, DIY repairs, exterior/interior decoration, home renovations)

	Walking pets/playing with pets (playing with cat/dog/pets, walking dog/pets)

Results

A total of 3614 participants were included in the analytic sample. Please refer to the main manuscript for participant characteristics.

Table A4 shows longitudinal trends in overall PA and each domain of PA. On average, overall PA declined by approximately 22 minutes/day between 10-11y and 12-13y (β=-22.0; 95% CI=-27.0, -17.0; p<0.001). Non-organized PA declined the most out of all domains between waves (β=-24.9; 95% CI=-28.8, -21.0; p<0.001). Active transport increased slightly between waves (β=1.8; 95% CI=0.4, 3.2; p=0.014). Trends in organized PA and active chores/work remained relatively stable. Figure A1 illustrates the average participation in each domain of PA at 10-11y and 12-13y.

Figure A1 Average participation in domains of PA by wave, with confidence intervals, weighted LSAC data (B Cohort)

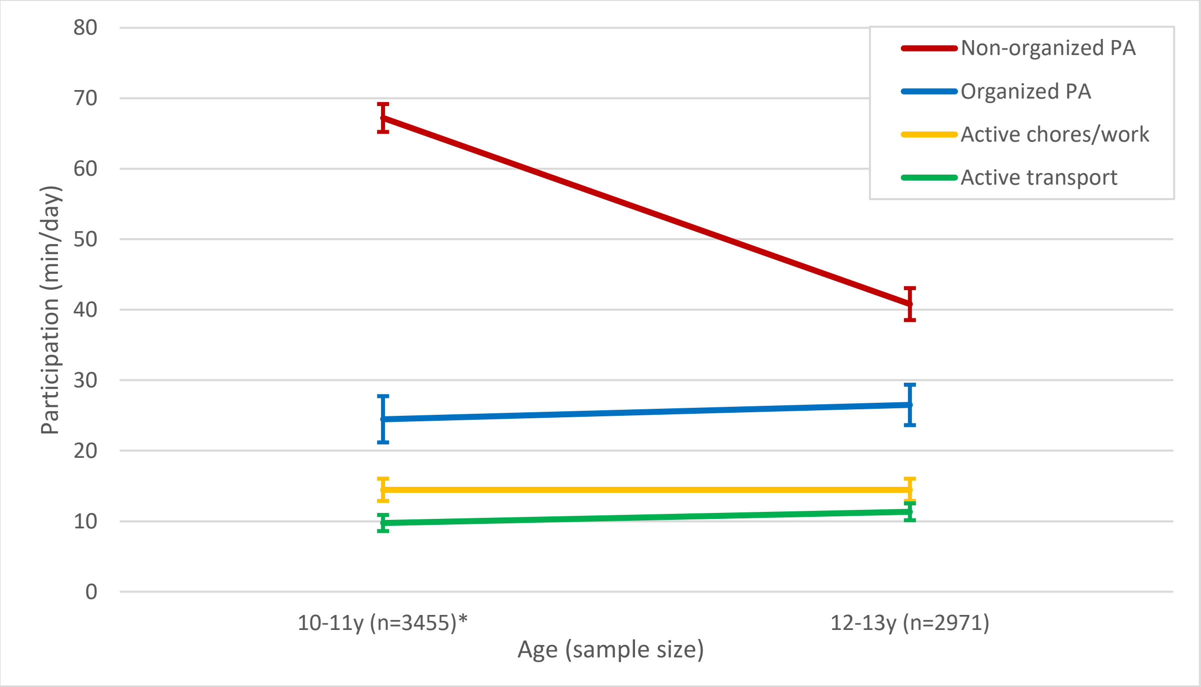

*One case was removed for active transport due to an implausibly high value.

Table A4 Participation in domains of PA (minutes/day) by wave, weighted LSAC data (B Cohort)

		Organized PA	Non-organized PA	Active transport	Active work/ chores	Overall PA
**PA domains, minutes/day**						
Wave 6 (n=3455)	Mean (SD)	24.5 (59.1)	67.2 (98.0)	9.8 (34.2)[a]	15.3 (40.4)	116.8 (122.8)
Wave 7 (n=2971)	Mean (SD)	26.5 (62.7)	40.8 (79.4)	11.3 (33.0)	14.5 (43.7)	93.4 (113.3)
**Unadjusted models** [b]						
Fixed effect - wave	β (95% CI)	2.0 (-0.6, 4.7)	-26.4 (-30.3, -22.5)	1.6 (0.1, 3.0)	-0.9 (-2.9, 1.1)	-23.5 (-28.4, -18.5)
	p value	0.13	<0.001	0.03	0.39	<0.001
**Adjusted models** [bc]						
Fixed effect - wave	β (95% CI)	1.4 (-1.3, 4.1)	-24.9 (-28.8, -21.0)	1.8 (0.4, 3.2)	-0.4 (-2.4, 1.7)	-22.0 (-27.0, -17.0)
	p value	0.32	<0.001	0.01	0.73	<0.001

PA = physical activity; LSAC = Longitudinal Study of Australian Children; SD = standard deviation; β = model coefficient; CI = confidence interval; W=wave

a. One case was removed for active transport due to an implausibly high value.

b. Multilevel mixed models (n=3614)

c. Adjusted for season and school attendance on the day of TUD completion

Comparison with previous research

These analyses revealed very similar results to previous research conducted using data from the Kindergarten (K) cohort of LSAC [4]. In particular, both B and K cohort data revealed a large decline in overall PA and non-organized PA between 10-11y and 12-13y. A small increase in active transport and a stable trend in organized PA were also observed between waves among both cohorts. The main difference between cohorts was in regard to active chores/work, as this domain remained stable between waves in B cohort data but increased slightly in K cohort data ($\beta=3.1$; 95% CI=1.6, 4.6; $p<0.001$) [4].

Conclusion

Despite some variation between LSAC cohorts, the overall pattern of large declines in non-organized PA and overall PA between 10-11y and 12-13y was confirmed using B cohort data. This paves the way for the investigation of subdomains of non-organized PA described in the main manuscript.

References (cited in main manuscript):

4. Kemp BJ, Parrish A, Batterham M, Cliff DP. Participation in domains of physical activity among Australian youth during the transition to adolescence: a longitudinal study. J Phys Act Health. 2020;17(3):278-86. doi: 10.1123/jpah.2018-0705.

13. Mohal J, Lansangan C, Gasser C, Taylor T, Renda J, Jessup K, et al. Growing Up in Australia: The Longitudinal Study of Australian Children – Data User Guide, Release 9C1. Melbourne: Australian Institute of Family Studies. 2021. Available from: https://doi.org/10.26193/BAA3N6.

17. Usback S. Wave 7 weighting and non-response (LSAC technical paper no. 20). Canberra: Commonwealth of Australia. 2018. Available from: https://growingupinaustralia.gov.au/sites/default/files/lsac_wave_7_weighting_and_non-response_paper_20.pdf.
