# Supplementary material for: Changes in subdomains of non-organized physical activity between childhood and adolescence in Australia: a longitudinal study
Source: Int J Behav Nutr Phys Act. 2022 Jun 25;19:73. doi: 10.1186/s12966-022-01311-2 (PMC9233835; doi:10.1186/s12966-022-01311-2)
Supplement: Supplementary file 2 — Additional file 2. Documentation of the process used to clean time-use data. This file provides further details about the process used to clean time-use data, resulting in the final subdomains of non-organized PA used in analysis. [file 12966_2022_1311_MOESM2_ESM.pdf]

Changes in subdomains of non-organized physical activity between childhood and adolescence in Australia: a longitudinal study

Additional file 2: Documentation of the process used to clean time-use data

Table B1: Time-use diary activity codes used as the initial foundation for domains of physical activity in the present study, Longitudinal Study of Australian Children (B Cohort, Waves 6 and 7)

**Domain of physical activity**	**Time-use diary activities (codes)**
Organised physical activity	Archery/shooting sports (team & individual) (401 & 411),<br>Athletics/gymnastics (team & individual) (402 & 412),<br>Fitness / gym / exercise (team & individual) (403 & 413),<br>Ball Sports (team & individual) (404 & 416),<br>Martial arts / Dancing (team & individual) (405 & 414),<br>Motor Sports/Roller Sports/Cycling (team & individual) (406 & 415), Water/Ice/Snow Sports (team & individual) (407 & 417),<br>Organised team sports and training other (team & individual) (408 & 418)
Non-organised physical activity*	Archery / Shooting sports (unstructured) (421),<br>Athletics / Gymnastics (unstructured) (422),<br>Fitness / Gym / Exercise (unstructured) (423),<br>Ball Sports (unstructured) (424),<br>Martial arts / Dancing (unstructured) (425),<br>Motor Sports / Roller Sports / Cycling (unstructured) (426),<br>Water / Ice / Snow Sports (unstructured) (427),<br>Non-organised PA (other) (original label: Unstructured active play Other (428))
Active transport	[Travel] by bike, scooter, skateboard etc. (911),<br>Travel by foot (901)
Active chores and work	Labourers and related workers (031),<br>Gardening / lawn mowing [paid] (041),<br>Apprenticeships/tradespersons (061),<br>Umpiring [paid] (082),<br>Car washing [paid] (083),<br>Cleaning/tidying (301),<br>Gardening (maintenance chores) (341),<br>Cleaning grounds/garage/shed/outside of house (chores) (342),<br>Pool care (chores) (344),<br>Design/Home Improvement (362),<br>Walking pets/playing with pets (430)
Other active activities	Active club activities (440)<br>Active activities not elsewhere classified (491)

*Note: the codes listed as part of this domain formed the initial subdomains of non-organized PA.

Table B2: Activities that were considered likely to be under 3.0 METs and were removed from PA domains in the present study, Longitudinal Study of Australian Children (B Cohort, Waves 6 and 7)

**Original domain**	**Text descriptions of activities that were removed from PA**	**PA compendia & METs (references)**
Organised PA	Billiards/pool (organised)	'Pool/billiards/snooker' – 2.5 METs [21]
	Boating (organised)	Author discussion [a]
	Car racing/speedway (organised)	'Riding in/driving a car' – 1.4 METs [21]
	Go Karting (organised)	Author discussion [b]
	Gyroplane flying (organised)	'Riding in a plane' – 1.4 METs [21]
	Hunting (with gun)/shotgun shooting (organised)	'Pistol shooting or trap shooting, standing' – 2.5 METs [22]
Non-organised PA	Billiards/Snooker/Pool/Eight-ball (unstructured)	'Pool/billiards/snooker' – 2.5 METs [21]
	Boating/Power boating (unstructured)	'Riding in/driving a car' – 1.4 METs [21]
	Darts (unstructured)	'Darts' – 2.5 METs [21]
	Driving for pleasure/scenic drive around	'Riding in/driving a car' – 1.4 METs [21]
	Go Karting (unstructured)	Author discussion [b]
	Motor sport (other) (unstructured)	Author discussion [b]
	Ride motorbike around	'Riding on a motorcycle / motor scooter' – 2.5 METs [21]
	Shooting sport (other) (unstructured)	'Pistol shooting or trap shooting, standing' – 2.5 METs [22]
Active transport	Segway (ride travel)	Author discussion [c]
Active chores/work	Bird (teaching to talk), Clock (adjust/set), House (unlock), Look for lost things, Pat the cat/dog/pets, Straighten up desk, Television (tune)	These activities were considered either 'sitting' (1.4 METs), 'standing' (1.8 METs) or 'walking – light effort' (2.9 METs) [21]
	Employment – mechanic's assistant	'Fixing things up (e.g. bike / toys / mechanical work on car etc)' – 2.5 METs [21]
	Put things away/pack up toys/pack up games	'Putting away or carrying groceries' – 2.5 METs [20]
	Toilet (clean)	'Scrubbing floors, on hands and knees, scrubbing bathroom, bathtub, light effort' – 2.0 METs [22]
Other active activities	Air show (participate), Attend athletics carnival/party/social gathering/sporting event/swimming carnival, Autograph (line up), Dinner (go out), Form (fill in), Free time, Heater (sit), Host party/visitors, Looking around/look at view/look out window, Looking at photos, Other free time (not further defined), Parent teacher interview, Presents (exchange/open), Put away/chain bike, Reminiscing, Sleep over, Socialising, Spa (in), Sunbathing, Trophy	These activities were considered either 'sitting' (1.4 METs), 'standing' (1.8 METs) or 'walking – light effort' (2.9 METs) [21]

	(receive), Visiting friend/relative, Waiting (general), Write in journal	
	Bird watching	'Bird watching, slow walk' – 2.5 METs [22]
	Camping/Holiday camp	'Camping - sitting' – 2.5 METs [21]

a. Boating was considered more likely to refer to being a passenger/driver in a motorised boat, rather than more active options such as canoeing or kayaking.
b. An equivalent compendium category could not be found. The category 'riding on a motorcycle or motor scooter' was used (2.5 METs) [21]
c. An equivalent compendium category could not be found. This activity was considered similar to 'Wii Balance (active video games)' (2.3-2.5 METs) [20]

Table B3: Activities that were reallocated to a different domain of PA in the present study, Longitudinal Study of Australian Children (B Cohort, Waves 6 and 7)

**Activities reallocated**	**Original domain (& subdomain)***	**New domain (& subdomain)***
Active club activities, active club meeting, bushwalking (club), Scouts/cubs/girl guides (active), active club activities (not further defined)	Active club activities	Organised PA
Organise dance / musical / party / play / performance / sporting event [a]	Active activities (not elsewhere classified)	Active chores/work
Participating in athletics carnival / swimming carnival / sports carnival / sports match	Active activities (not elsewhere classified)	Organised PA
Slip n Slide	Active activities (not elsewhere classified)	Non-organised PA (Non-organised PA (other))
Tent (set/pack up)	Active activities (not elsewhere classified)	Active chores/work
Wood chopping (unstructured) [b]	Non-organised PA (Non-organised PA (other))	Active chores/work

*Subdomains included for non-organised PA only

a. The authors reasoned that these activities could involve a range of tasks including putting out chairs, building sets, putting up decorations, etc. On balance, these activities were regarded as similar to 'standing tasks, light effort (e.g., bartending, store clerk, assembling, filing, duplicating, librarian, putting up a Christmas tree, standing and talking at work, changing clothes when teaching physical education, standing)' (3.0 METs) [22].

b. When occurring in an unstructured context, wood chopping was considered more likely to contribute to household tasks (active chores/work) than to occur for its own sake (non-organised PA).

Table B4: Activities that were reallocated to a different subdomain within non-organised PA in the present study, Longitudinal Study of Australian Children (B Cohort, Waves 6 and 7)

**Activities reallocated**	**Original subdomain**	**New subdomain**	**Rationale**
Archery	Archery / Shooting sports (unstructured)	Non-organised PA (other)	The ‘Archery / Shooting sports (unstructured)’ subdomain was collapsed due to having <10 instances of non-zero values in both W6 and W7.
Yabbying	Archery / Shooting sports (unstructured)	Non-organised PA (other)	
Trampolining	Athletics / Gymnastics (unstructured)	Non-organised PA (other)	In the unstructured/non-organised context, this activity was deemed more likely to occur as part of active play (e.g. using backyard trampolines for fun) rather than as part of athletics/gymnastics training.
Skipping / rope skipping	Fitness / Gym / Exercise (unstructured)	Non-organised PA (other)	At 10-11y and 12-13y, these activities were considered more likely to occur as part of active play (e.g. for fun) rather than for intentional exercise.
Tenpin bowling / lawn bowls / carpet bowls	Non-organised PA (other)	Ball Sports (unstructured)	These activities were considered ball sports because ‘Tenpin bowling’ and ‘bowls’ are recognised sports on the SportAus Australian Sport Directory [47].
Ultimate Frisbee (unstructured)	Non-organised PA (other)	Ball Sports (unstructured)	This activity is a recognised sport on the SportAus Australian Sport Directory [47]. Although a ball is not involved, the activity is otherwise very similar to other ‘invasion’ sports in this category such as Australian-rules football and netball [48].
Skate park (unstructured)	Non-organised PA (other)	Roller Sports / Cycling (unstructured)	The authors determined that this was likely shorthand for ‘skating at a skate park’.
Non-motorised scooter (ride unstructured)	Water/ice/snow sports (unstructured)	Roller Sports / Cycling (unstructured)	The authors believed that the original code was most likely a coding error.
Fishing	Water/ice/snow sports (unstructured)	Non-organised PA (other)	Fishing was considered more similar to ‘yabbying’ than other activities in this subdomain.
Walking (unstructured exercise)	Non-organised PA (other)	Fitness / Gym / Exercise (unstructured)	Adjusted because the activity seemed to occur for the purpose of ‘exercise’.

Table B5: Final subdomains of non-organised PA included in the present study, Longitudinal Study of Australian Children (B Cohort, Waves 6 and 7)

Subdomain of non-organised PA	Included activities	PA compendia & METs (references)
**Athletics/gymnastics**	Acrobatics	'Gymnastics - moderate effort' – 4.0 METs [21]
	Cheerleading	'Cheerleading, gymnastic moves, competitive' – 6.0 METs [22]
	Gymnastics	'Gymnastics - moderate effort' – 4.0 METs [21]
**Ball sports**	American football, Australian Rules football/AFL, Football (other)	'Football (Australian/American) - moderate effort' – 8.8 METs [21]
	Badminton	'Badminton - moderate effort' – 4.5 METs [21]
	Baseball	'Baseball - moderate effort' – 5.0 METs [21]
	Basketball	'Basketball – game' – 6.2-6.4 METs [20]
	Carpet bowls/lawn bowls	'Lawn bowls' – 3.0 METs [21]
	Golf	'Golf - moderate effort' – 4.3 METs [21]
	Handball	'Handball' – 5.6-5.7 METs [20]
	Netball	'Netball - moderate effort' – 8.2 METs [21]
	Outdoor cricket	'Cricket - moderate effort' – 3.5 METs [21]
	Outdoor hockey	'Hockey (field) - moderate effort' – 8.0 METs [21]
	Outdoor soccer	'Soccer – game' – 8.1-8.4 METs [20]
	Oztag	'Touch football - moderate effort' – 8.8 METs [21]
	Putt-putt golf/minigolf	'Golf – game (mini golf)' – 3.9 METs [20]
	Rugby league	'Rugby league - moderate effort' – 8.8 METs [21]
	Rugby union	'Rugby union - moderate effort' – 8.8 METs [21]
	Softball, T ball	'Softball or t-ball - moderate effort' – 5.0 METs [21]
	Table tennis/ping pong	'Table tennis' – 4.2 METs [20]
	Tennis	'Tennis practice and games' – 6.3-6.5 METs [20]
	Tenpin bowling	'Bowling – game' – 5.4-5.6 METs [20]
	Touch football	'Touch football - moderate effort' – 8.8 METs [21]
	Ultimate Frisbee	'Ultimate Frisbee' – 5.8-5.9 METs [20]
	Volleyball/beach volleyball	'Volleyball' – 5.1-5.2 METs [20]
	Non-organised ball sports (other)	Author discussion [a]
	Bike riding/cycling, BMXing/mountain biking	'Riding a bike – self paced' – 5.3-5.8 METs [20]

**Cycling/roller/motor sports**	Dirt/trail biking motorised	'Moto-cross, off-road motor sports, all-terrain vehicle, general' – 4.0 METs [22]
	Roller blading/skating	'Rollerblading' – 5.2-5.3 METs [20]
	Scooter (ride unstructured)	'Riding scooter' – 5.6-6.2 METs [20]
	Skateboarding/Skate park	'Riding a skateboard - moderate effort' – 5.0 METs [21]
	Non-organised cycling/roller sports (other)	Author discussion [a]
**Fitness/gym/exercise**	Calisthenics	'Calisthenics – moderate effort' – 5.8 METs [21]
	Circuits	'Circuit training, moderate effort' – 4.3 METs [22]
	Exercise biking	'Riding a bike – self paced' – 5.3-5.8 METs [20]
	Exercising, Gym workouts	Author discussion [a]
	Jogging, Running for exercise,	'Running/jogging - moderate effort' – 8.5 METs [21]
	Treadmill activities	At least 'walk self-paced casual' – 3.9-4.2 METs [20]
	Walking (unstructured exercise)	At least 'walk self-paced casual' – 3.9-4.2 METs [20]
	Weightlifting	Author discussion [b]
**Martial arts/dancing**	Boxing	'Boxing - punching bag and gloves' – 5.0 METs [20]
	Dancing (other)	'Dancing (general) - moderate effort' – 5.5 METs [21]
	Martial arts (other)	'Karate/martial arts/judo /kick boxing - moderate effort' – 10.0 METs [21]
	Wrestling	'Wrestling with mates' – 4.0 METs [21]
	Non-organised martial arts/dancing (other)	Author discussion [a]
**Water/ice/snow sports**	Bodyboarding	'Surfing (body or board) - moderate effort' – 5.0 METs [21]
	Canoeing	'Rowing/canoeing - moderate effort' – 7.0 METs [21]
	Ice skating	'Ice skating - moderate effort' – 7.0 METs [21]
	Sailing	'Sailing/boating - moderate effort' – 3.0 METs [21]
	Snowboarding	'Skiing, downhill, alpine or snowboarding, moderate effort, general, active time only' – 5.3 METs [22]
	Snow skiing	'Skiing' – 5.8-6.0 METs [20]
	Surf sport/Surfing	'Surfing (body or board) - moderate effort' – 5.0 METs [21]
	Swimming	'Swimming – self-selected pace' – 8.9-9.1 METs [20]
	Water-skiing	'Water skiing - moderate effort' – 6.0 METs [21]
	Non-organised water/ice/snow sports (other)	Author discussion [a]
**Active play**	Flying disc games/Boomerang throwing	'Frisbee (general) - moderate effort' – 3.0 METs [21]

	Flying kites	Author discussion [c]
	Hide and seek	'Hide and seek' – 4.0 METs [21]
	Hula hoop	'Free play (basketball, rope, hoop, climb, ladder, frisbee' – 5.9-6.0 METs [20]
	Kicking ball, Ball games	'Ball games - bouncing, kicking, dribbling ball, reaction ball (moderate intensity)' – 6.2-6.3 METs [20]
	Play on play equipment (Playground/Play on play equipment, Going to the park)	'Playground equipment (e.g. monkey bars) - moderate effort' – 5.0 METs [21]
	Rope skipping, Skipping	'Jump rope' – 7.1-7.2 METs [20]
	Slip n slide	Author discussion [d]
	Throwing ball against wall	'Catch/throw ball' – 4.1 METs [20]
	Tips, Chasing/running around	'Playing tag – moderate' – 6.3-6.4 METs [20]
	Trampolining	'Trampoline' – 7.1-7.3 METs [20]
	Water fight	'Free play (basketball, rope, hoop, climb, ladder, frisbee' – 5.9-6.0 METs [20]
	Unstructured active play (not further defined).	Author discussion [a]
**Other outdoor /nature PA**	Archery (unstructured)	'Archery' – 3.5 METs [21]
	Bush walking	'Hiking' – 6.0-6.1 METs [20]
	Exploring/Going sightseeing	'Walking - self paced casual' – 3.9-4.2 METs [20]
	Fishing	'Fishing' (3.0 METs) [21]
	Horse riding	'Horseback riding - moderate effort' – 4.0 METs [21]
	Yabbying	Author discussion [e]

a. Authors agreed to include 'other' categories and general 'exercise' categories as PA based on face value.
b. The authors decided to include this as PA because the Australian PA guidelines include recommendations for muscle and bone strengthening activities for children and young people (5-17y) [49].
c. An equivalent compendium category could not be found. The authors reasoned that 'flying kites' during childhood is likely to involve running to get the kite in the air which is repeated due to frequent crashes. This activity was considered at least as active as 'hide and seek' (4.0 METs) [21].
d. An equivalent compendium category could not be found. 'Slip n slide' was considered similar to 'bobsled toboggan luge – light effort' (5.3 METs) [21].
e. An equivalent compendium category could not be found. 'Yabbying' was considered at least as active as 'fishing' (3.0 METs) [21].

References (cited in main manuscript):

20. Butte NF, Watson KB, Ridley K, Zakeri IF, McMurray RG, Pfeiffer KA, et al. A youth compendium of physical activities: activity codes and metabolic intensities. Med Sci Sports Exerc. 2018;50(2):246. doi: 10.1249%2FMSS.0000000000001430

21. Ridley K, Ainsworth BE, Olds TS. Development of a compendium of energy expenditures for youth. Int J Phys Act Beh Nutr. 2008;5(1):1-8. doi: 10.1186/1479-5868-5-45

22. Ainsworth BE, Haskell WL, Herrmann SD, Meckes N, Bassett DR, Tudor-Locke C, et al. 2011 Compendium of Physical Activities: a second update of codes and MET values. Med Sci Sports Exerc. 2011;43(8):1575-81. doi: 10.1249/MSS.0b013e31821ece12

References (not cited in main manuscript):

47. Sports Australia. Australian Sports Directory n.d. [Available from: https://www.sportaus.gov.au/australian_sports_directory]

48. Griggs G. ‘Just a sport made up in a car park?’: The ‘soft’ landscape of Ultimate Frisbee. Soc Cult Geogr. 2009;10(7):757-70. doi: 10.1080/14649360903205124

49. Commonwealth of Australia | Department of Health. Physical activity and exercise guidelines for all Australians 2021 [Available from: https://www.health.gov.au/health-topics/physical-activity-and-exercise/physical-activity-and-exercise-guidelines-for-all-australians/for-children-and-young-people-5-to-17-years.
