# Supplementary material for: Changes in subdomains of non-organized physical activity between childhood and adolescence in Australia: a longitudinal study
Source: Int J Behav Nutr Phys Act. 2022 Jun 25;19:73. doi: 10.1186/s12966-022-01311-2 (PMC9233835; doi:10.1186/s12966-022-01311-2)

**Changes in subdomains of non-organized physical activity between childhood and adolescence in Australia: a longitudinal study**

**Additional file 3: Participation in subdomains of non-organized PA at 10-11y and 12-13y, showing mean participation (min/day) and 95% confidence intervals (weighted LSAC data, B cohort)**

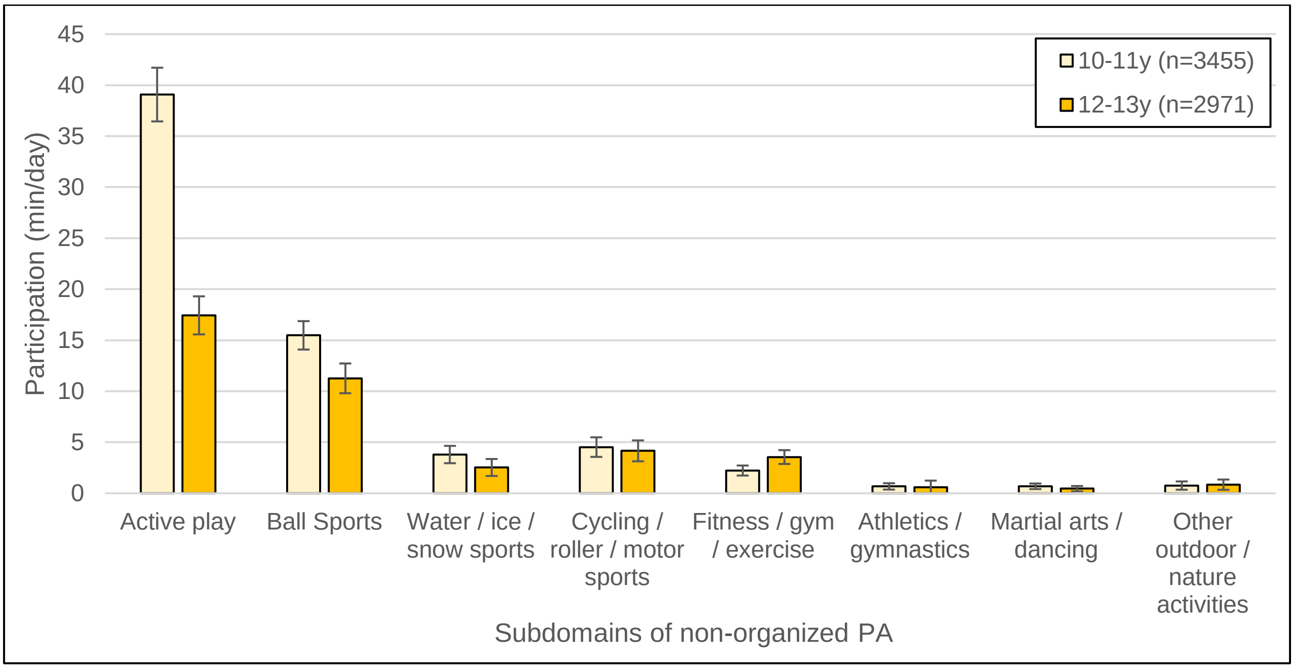

Supplement: Supplementary file 3 — Additional file 3. Participation in subdomains of non-organized PA at 10-11y and 12-13y, showing mean participation (min/day) and 95% confidence intervals (weighted LSAC data, B cohort). This file provides an additional data visualisation of the changes in mean participation in subdomains of non-organized PA at both waves, including 95% confidence intervals. [file 12966_2022_1311_MOESM3_ESM.pdf]
