# Supplementary material for: Changes in subdomains of non-organized physical activity between childhood and adolescence in Australia: a longitudinal study
Source: Int J Behav Nutr Phys Act. 2022 Jun 25;19:73. doi: 10.1186/s12966-022-01311-2 (PMC9233835; doi:10.1186/s12966-022-01311-2)
Supplement: Supplementary file 4 — Additional file 4. All potential moderation effects between wave and sex on subdomains of non-organized PA (minutes/day), weighted LSAC data, B Cohort. This file provides the results from all post-hoc models used to test interactions between wave and sex for each subdomain of non-organized PA. [file 12966_2022_1311_MOESM4_ESM.pdf]

Changes in subdomains of non-organized physical activity between childhood and adolescence in Australia: a longitudinal study

Additional file 4: All potential moderation effects between wave and sex on subdomains of non-organized PA (minutes/day), weighted LSAC data, B Cohort

**Subdomains of non-organized PA (min/day)**	**Potential moderation effect: wave*sex (girls) [ab]**	
	**β (95% CI)**	**p value**
Active play	-8.2 (-13.9, -2.4)	0.006
Ball sports	2.2 (-1.4, 5.8)	0.237
Water/ice/snow sports	0.8 (-1.6, 3.1)	0.520
Cycling/roller/motor sports	-2.5 (-5.2, 0.1)	0.063
Fitness/gym/exercise	0.6 (-1.1, 2.3)	0.489
Athletics/gymnastics	0.4 (-1.1, 1.9)	0.602
Martial arts/dancing	0.5 (-0.3, 1.2)	0.194
Other outdoor/nature activities	-1.0 (-2.3, 0.2)	0.109

PA = physical activity; LSAC = Longitudinal Study of Australian Children; β = model coefficient; CI = confidence interval

a. Multilevel mixed models (n=3614), adjusted for season and school attendance on the day of TUD completion

b. Reference category: boys
