# Supplementary material for: Changes in subdomains of non-organized physical activity between childhood and adolescence in Australia: a longitudinal study
Source: Int J Behav Nutr Phys Act. 2022 Jun 25;19:73. doi: 10.1186/s12966-022-01311-2 (PMC9233835; doi:10.1186/s12966-022-01311-2)
Supplement: Supplementary file 5 — Additional file 5. STROBE Statement. Completed STROBE checklist as requested in submission guidelines. [file 12966_2022_1311_MOESM5_ESM.pdf]

STROBE Statement—Checklist of items that should be included in reports of ***cohort studies***

	Item No	Recommendation	Page No. (tracked changes accepted)	Relevant text from manuscript (if appropriate)
**Title and abstract**	1	(*a*) Indicate the study's design with a commonly used term in the title or the abstract	1	"Changes in subdomains of non-organized physical activity between childhood and adolescence in Australia: *a longitudinal study"*
		(*b*) Provide in the abstract an informative and balanced summary of what was done and what was found	2	–
**Introduction**				
Background/rationale	2	Explain the scientific background and rationale for the investigation being reported	2-5	–
Objectives	3	State specific objectives, including any prespecified hypotheses	5	"This study aimed to investigate two research questions: (1) How does the duration of subdomains of non-organized PA change between late childhood (10-11y) and early adolescence (12-13y) in Australia; and (2) Are these changes moderated by sex?"
**Methods**				
Study design	4	Present key elements of study design early in the paper	5	"This study used data from the Baby (B) cohort of the Longitudinal Study of Australian Children (LSAC), a longitudinal research project managed by the Australian Department of Social Services (DSS)"
Setting	5	Describe the setting, locations, and relevant dates, including periods of recruitment, exposure, follow-up, and data collection	5-6	"LSAC includes two cohorts of participants who were recruited in 2004. The Kindergarten (K) cohort were recruited at 4-5y and the Baby (B) cohort were aged 0-12 months at baseline"<br>"Both cohorts were designed to be nationally-representative samples of the Australian population, with stratified selection being used to ensure proportional inclusion of members of each Australian state and territory across major city and regional/remote areas [16]. Both cohorts have been followed-

				up every two years to date."<br>"B cohort participants were 10-11y in 2014 (Wave 6) and 12-13y in 2016 (Wave 7)"
Participants	6	(*a*) Give the eligibility criteria, and the sources and methods of selection of participants. Describe methods of follow-up	5-6	"The Kindergarten (K) cohort were recruited at 4-5y and the Baby (B) cohort were aged 0-12 months at baseline [13]. Participants in both cohorts were recruited from the Australian Medicare database via a two-stage clustered design, involving the random selection of postcodes then families [13]."<br>"Both cohorts have been followed-up every two years to date, with a variety of follow-up methods being employed including mail, telephone and in-person visits [13]."
		(*b*) For matched studies, give matching criteria and number of exposed and unexposed	N/A	N/A
Variables	7	Clearly define all outcomes, exposures, predictors, potential confounders, and effect modifiers. Give diagnostic criteria, if applicable	7-9	[Physical activity measures are defined on pages 6-8 and in Additional file 2].<br>[Potential effect modifiers and confounding variables are defined on page 9].
Data sources/ measurement	8*	For each variable of interest, give sources of data and details of methods of assessment (measurement). Describe comparability of assessment methods if there is more than one group	7-9	[Time use diaries used to measure physical activity are described on pages 6-8].<br>[Details of other measures given on page 9]
Bias	9	Describe any efforts to address potential sources of bias	9	"Models were also controlled for two potential confounding variables: season of measurement and whether the child attended school on the day of TUD completion (yes/no)."<br>"The LSAC Wave 1 and 6 population data weights were applied to reduce bias associated with attrition and improve the representativeness of the data."
Study size	10	Explain how the study size was arrived at	10	"A total of 3614 participants provided complete TUD data for at least one wave and were included in the models (96% of the total W6 sample). Among these, 3455 participants provided complete TUD data for W6 and 2971 participants provided complete TUD data for W7"

Quantitative variables	11	Explain how quantitative variables were handled in the analyses. If applicable, describe which groupings were chosen and why	9	[Under the heading 'Analysis']
Statistical methods	12	(*a*) Describe all statistical methods, including those used to control for confounding	9	[Under the heading 'Analysis']
		(*b*) Describe any methods used to examine subgroups and interactions	9	"post-hoc models tested interactions between wave and sex"
		(*c*) Explain how missing data were addressed	9	"Missing data for school attendance was imputed using the 'school lessons' code in the TUD (it was assumed that children attended school if this code was used)."<br>"All available data were included in models, as multilevel modelling does not require complete cases for every time-point [24]."
		(*d*) If applicable, explain how loss to follow-up was addressed	9	"The LSAC Wave 1 and 6 population data weights were applied to reduce bias associated with attrition and improve the representativeness of the data."
		(*e*) Describe any sensitivity analyses	N/A	N/A
**Results**				
Participants	13*	(a) Report numbers of individuals at each stage of study—eg numbers potentially eligible, examined for eligibility, confirmed eligible, included in the study, completing follow-up, and analysed	6, 9	"A total of 3764 participants took part in the W6 face-to-face interview and 3614 of these participants provided complete TUD data for at least one wave and were included in the models (96% of the total W6 sample). Among these, 3455 participants provided complete TUD data for W6 and 2971 participants provided complete TUD data for W7."
		(b) Give reasons for non-participation at each stage	10	"A total of 3764 participants took part in the W6 face-to-face interview and 3614 of these participants provided complete TUD data for at least one wave and were included in the models (96% of the total W6 sample)."
		(c) Consider use of a flow diagram	N/A	N/A
Descriptive data	14*	(a) Give characteristics of study participants (eg demographic, clinical, social) and information on exposures and potential	10<br>Table 2	"A similar proportion of those with valid TUD data at W7 were male compared with 12-13 year-olds in the 2016

		confounders	Additional File 1	Australian Census (51.0% at W7 versus 51.4% nationally) [25]. The analytic sample somewhat overrepresented participants who lived in regional or remote areas (36.3% at W7 versus 30.4% nationally) and underestimated Aboriginal and Torres Strait Islander participants (2.3% at W7 versus 5.1% nationally) and those who spoke languages other than English at home (8.8% at W7 versus 16.9% nationally) [25].
		(b) Indicate number of participants with missing data for each variable of interest	Table 2	Footnotes to Table 2
		(c) Summarise follow-up time (eg, average and total amount)	Table 2	"Of the participants with valid data in both waves, the average time between waves was 24.5 months (SD=3.0, n=2812)."
Outcome data	15*	Report numbers of outcome events or summary measures over time	Table 3	Column: "Participation, min/day Mean (SD)"
Main results	16	(*a*) Give unadjusted estimates and, if applicable, confounder-adjusted estimates and their precision (eg, 95% confidence interval). Make clear which confounders were adjusted for and why they were included	Table 3	Columns: "Unadjusted models – fixed effect of wave", "Adjusted models – fixed effect of wave".<br>Footnote b.
		(*b*) Report category boundaries when continuous variables were categorized	N/A	N/A
		(*c*) If relevant, consider translating estimates of relative risk into absolute risk for a meaningful time period	N/A	N/A
Other analyses	17	Report other analyses done—eg analyses of subgroups and interactions, and sensitivity analyses	11<br>Additional file 4	"Active play was the only subdomain of non-organized PA that exhibited a notable moderation effect by sex. As shown in Fig 3, a steeper decline between 10-11y and 12-13y occurred among girls compared with boys (β= –8.2, 95% CI= –13.9, –2.4; p=0.006). Additional file 4 provides the results of all moderation tests."
**Discussion**				
Key results	18	Summarise key results with reference to study objectives	10-11	–
Limitations	19	Discuss limitations of the study, taking into account sources of potential bias or imprecision. Discuss both direction and	14-15	–

		magnitude of any potential bias		
Interpretation	20	Give a cautious overall interpretation of results considering objectives, limitations, multiplicity of analyses, results from similar studies, and other relevant evidence	11-14	–
Generalisability	21	Discuss the generalisability (external validity) of the study results	14-15	"In addition, although the chosen population weight was considered the best available option, it did not account for non-response beyond Wave 6. This resulted in some population groups being under- or over-represented in the sample, which was only partially attenuated through the use of data weights. Participants who spoke languages other than English at home were particularly under-represented, which may be due to LSAC being a 'closed' longitudinal study. No new participants have been recruited to LSAC since Wave 1, which means immigrants arriving in Australia after 2004 have not been included. These factors should be considered when applying the results of this study in the Australian context or elsewhere.
**Other information**				
Funding	22	Give the source of funding and the role of the funders for the present study and, if applicable, for the original study on which the present article is based	21	"BK is the recipient of a Prioritising Emerging Research Leaders (PERL) Fellowship, provided by the University of Wollongong (UOW). This funding body did not influence the conduct of this study in terms of design, analysis and interpretation or writing."

*Give information separately for exposed and unexposed groups.

Note: An Explanation and Elaboration article discusses each checklist item and gives methodological background and published examples of transparent reporting. The STROBE checklist is best used in conjunction with this article (freely available on the Web sites of PLoS Medicine at http://www.plosmedicine.org/, Annals of Internal Medicine at http://www.annals.org/, and Epidemiology at http://www.epidem.com/). Information on the STROBE Initiative is available at http://www.strobe-statement.org.
